# Efficacy and safety of 15 mA transcranial alternating current stimulation (tACS) in the therapy of treatment-resistant depression with increased inflammatory activity: Study protocol for a randomized, double-blind, sham-controlled trial

**DOI:** 10.1371/journal.pone.0356611

**Published:** 2026-08-27

**Authors:** Xuguang An, Jin Zhang, Xiaofei Hou, Chenghao Yang

**Affiliations:** Tianjin Anding Hospital, Tianjin Mental Health Center, Hexi District, Tianjin, China; Mae Fah Luang University School of Anti Aging and Regenerative Medicine, THAILAND

## Abstract

**Background and Objectives:**

Treatment-resistant depression (TRD) poses a significant clinical challenge, with traditional antidepressant therapies often ineffective. Elevated peripheral inflammation has emerged as a potential biological subtype of TRD, highlighting the need for novel treatments. Transcranial alternating current stimulation (tACS) has garnered attention for its potential antidepressant effects, particularly through its impact on glial cells, which are involved in neuroinflammation and neuronal interactions. This study aims to assess the efficacy and safety of high-intensity (15 mA) tACS in TRD patients with elevated inflammatory activity.

**Methods:**

This randomized, double-blind, sham-controlled trial will recruit 70 participants based on C-reactive protein levels (0.85–10 mg/L) and Dutch Method for Staging Therapy-Resistant Depression scores (DM-TRD, > 12.5). The study involves a 4-week tACS/sham intervention followed by an 8-week follow-up. The primary endpoint includes changes in the Montgomery-Åsberg Depression Rating Scale (MADRS) scores from baseline to week 4. Secondary endpoints include MADRS scores at week 2, Self-rating depression and anxiety scale scores, response and remission rates, and the presence of epileptiform activity. It will explore the association between changes in MADRS scores and alterations in EEG parameters and/or concentrations of peripheral biomarkers. Safety assessments will monitor adverse events, vital signs, and ECG parameters, with serious adverse events defined by specific medical conditions.

**Discussion:**

Understanding the contribution of inflammatory processes to the heterogeneity of depression may provide important insights into its underlying pathophysiological mechanisms and facilitate the identification of novel, mechanism-based therapeutic targets. In this context, the present study aims to explore the potential for the development of more effective, personalized treatment strategies tailored to depressive patients.

**Trial registration:**

At ClinicalTrials.gov (NCT06812923) on 02 February 2025. **Protocol version**: 15mA tACS 1.0

## Introduction

Major depressive disorder (MDD) remains a significant clinical challenge, with approximately 30% of patients developing treatment-resistant depression (TRD) despite both initial and subsequent treatment attempts [1]. The intricate nature of TRD is underscored by its association with a range of biological, genetic, and environmental factors [2], which complicate treatment strategies and contribute to a substantial disease burden at both the individual and societal levels. Among them, inflammatory cytokines have been increasingly implicated in the pathophysiology of MDD, particularly in relation to treatment response. For example, a systematic review and meta-analysis reveal that MDD patients who responded to antidepressant treatment had lower baseline levels of interleukin-8 (IL-8) than non-responders did. Moreover, treatment significantly decreases tumor necrosis factor-alpha (TNF-α) levels only in responders [3]. In our own study, patients with TRD who presented increased C-reactive protein (CRP) levels (0.85–10 mg/L) and baseline Dutch Method for Staging Therapy-Resistant Depression (DM-TRD) scores greater than 12.5 presented a more favorable response to a combination of antidepressants and N-acetylcysteine (NAC), an agent with anti-inflammatory properties, than did those receiving antidepressants with a placebo (NCT02972398) [4]. These findings suggest that inflammatory markers not only serve as potential predictors of treatment response but also represent promising targets for novel therapeutic interventions.

Transcranial alternating current stimulation (tACS) is a noninvasive neuromodulation technique that has shown potential in the treatment of MDD. Its therapeutic effects are thought to occur primarily through the entrainment of neuronal oscillations, which may directly influence synaptic plasticity and neuronal connectivity, both of which are central to MDD pathology [5]. One study showed that combining high-intensity (15 mA) tACS with antidepressants represents a viable and promising strategy for managing MDD, possibly through a mechanism involving reduced alpha power in the left frontal lobe [6]. Neurons are intricately connected with glial cells, which not only provide structural support but also actively participate in synaptic transmission and plasticity. For example, astrocytes release gliotransmitters that modulate synaptic activity, contributing to neuronal health and function [7]. Similarly, microglia interact with neurons to regulate physiological processes critical for brain development, including synapse formation and circuit refinement [8]. Notably, the role of glial cells, particularly astrocytes and microglia, in neuroinflammation is increasingly recognized as pivotal in MDD. Dysregulation of microglial activity has been linked to the exacerbation of depressive symptoms through the release of inflammatory cytokines and altered synaptic plasticity [9]. The modulation of microglial activity via external stimuli has been shown to alleviate neuroinflammation and improve depressive-like behaviors [10]. However, to date, few studies have explored the efficacy of tACS in the treatment of TRD or investigated the underlying mechanisms involving glial cells and neuroinflammatory processes.

In recent years, increasing evidence has supported the view that depression is not a homogeneous disorder with a single etiology but rather a heterogeneous syndrome driven by multiple pathophysiological mechanisms [11]. Stratifying TRD patients on the basis of specific biological characteristics may facilitate the identification of subgroups that are more responsive to particular treatment approaches, thereby promoting the development of personalized therapies. Notably, elevated peripheral inflammation has emerged as a potential biomarker for defining a biologically distinct TRD subtype characterized by inflammation-related pathophysiology [12]. Thus, targeting inflammatory processes in this subgroup may offer significant clinical benefits. The present study aims to evaluate the efficacy and safety of high-intensity (15 mA) tACS in individuals with TRD and elevated inflammatory activity. In addition to assessing reductions in depressive symptoms following the intervention, the safety and tolerability of the treatment will be evaluated. Furthermore, the exploratory analysis will focus on changes in peripheral biomarkers, including inflammatory cytokines and indicators of glial cell activity. It will also combine electroencephalographic (EEG) measures, such as power spectral density and phase synchrony, to provide preliminary insights into the potential neurophysiological mechanisms underlying the therapeutic effects of tACS.

## Materials and methods

### Study design

This is a double-blind, randomized, sham stimulation-controlled antidepressant augmentation trial. All the participants are randomly divided into two groups: “antidepressant + tACS” (N = 35) or “antidepressant + sham” (N = 35).

### Duration

The total study duration is 12 weeks (4 weeks of treatment and 8 weeks of follow-up). The blood samples, EEG, and scale assessments will be collected at multiple time points, including baseline, post-baseline, post-intervention, and post-follow up. See Table 1 for details.

**Table 1:** List of time points for sampling/assessments.

Time Point	Definition	Evaluation
Baseline	The day before 4-week intervention	Basic information collection, Scale assessments (not BBI)
Post baseline	The day following baseline	Blood sampling, EEG, Physical examination
Post intervention 1	The day of 11th-session intervention	Scale assessments except for DM-TRD and BBI
Post intervention 2	The day following the 4-week intervention	Scale assessments except for DM-TRD and BBI, Blood sampling, EEG, Physical examination
Post follow-up	The end of the 8-week follow-up	Scale assessments except for DM-TRD

### Setting

Treatment will be given at the Neuromodulation Center of Tianjin Anding Hospital. Information collection, scale assessment, and blood sampling are all conducted in the inpatient/outpatient department of Tianjin Anding Hospital.

### Population

The study population will consist of a sample of 70 patients with TRD and elevated inflammatory activity recruited from the outpatient and inpatient departments of Tianjin Anding Hospital. The recruitment of participants has been ongoing since January 1, 2025, and is expected to be completed by December 31, 2027. To date, 36 participants have been recruited and no serious adverse reactions have been reported.

### Ethics approval and consent to participate

The study protocol received approval from the Medical Ethics Committee of Tianjin Anding Hospital (Approval No. LSKD2024−69). Prior to participation, all participants are required to provide written informed consent regarding the study’s objectives and procedures. In cases where participants lack the capacity to provide consent, their legal guardians will sign the informed consent on their behalf.

### Inclusion criteria

Age between 18 and 55 years; diagnosis of MDD on the basis of a structured clinical interview conducted by a psychiatrist using the Diagnostic and Statistical Manual of Mental Disorders, Fifth Edition (DSM-5); Montgomery-Åsberg Depression Rating Scale (MADRS) total score of 20 or above; CRP levels between 0.85 and 10 mg/L; DM-TRD scores > 12.5; stable use of therapeutic medications (antidepressants, antipsychotics) for at least 2 weeks during the current depressive episode; and ability to understand and sign the informed consent form.

### Exclusion criteria

A history of manic episode; use of a stabilizer; use of antipsychotic medication at more than half of the maximum dosage recommended in the instruction; current or past history of seizures, epilepsy, hydrocephalus, central nervous system tumors, acute brain injuries, or infections; significant suicide risk; receiving neuromodulation treatment (modified electroconvulsive therapy, MECT; transcranial magnetic stimulation, TMS; transcranial direct current stimulation, tDCS; or tACS, etc.) within the past 2 months prior to enrollment; pregnant or breastfeeding women; any severe organic disease or patients in an unstable state due to organic disease; use of anti-inflammatory drugs for more than 7 days in total within the last 2 months or use of immunosuppressive drugs, such as corticosteroids; chronic infectious or autoimmune diseases, such as lupus, colitis, hepatitis, etc.; and history of substance dependence or abuse.

### Sample size calculation

The primary outcome is the change in MADRS score from baseline to week 4. Since the primary analysis will employ a linear mixed‑effects model for repeated measures (MMRM), sample size estimation was based on the repeated measures analysis of variance procedure, which accounts for the longitudinal design with a compound symmetry correlation structure. Although several randomized controlled trials have confirmed the efficacy of tACS combined with antidepressants for depression [6,13], no tACS study has specifically targeted TRD with elevated inflammatory activity. To inform our parameter assumptions, we drew on related neurostimulation literature. A pooled analysis reported mean MADRS reductions of 7.0 for rTMS and 7.6 for tDCS [14]. A retrospective tDCS study in TRD observed a mean MADRS reduction of approximately 14.9 points from baseline to post‑treatment [15]. A pilot tACS RCT for MDD reported a between‑group difference of approximately 7.8 points at the 2‑week follow‑up [16]. To ensure a conservative estimate, we set the target treatment effect at 7 points, which exceeds the established minimal clinically important difference (MCID) of 6 points for TRD [17]. The pooled standard deviation (SD) of the MADRS change was estimated as 10.0, based on the same sources, where reported SDs ranged from 7.2 to 11.8 [14–16]. A within‑subject autocorrelation coefficient of ρ = 0.5 was assumed, a conservative default in longitudinal sample size calculations. Using the repeated measures procedure in PASS 2021 with a two‑sided significance level (α) of 0.05, power (1‑β) of 0.80, δ = 7, σ = 10.0, and ρ = 0.5, the required effective sample size was 30 participants per group. We assumed a dropout rate of 15%, and the results indicated that 35 participants would be required for each group, yielding a total sample size of 70.

### Strategy for participant enrolment

Firstly, participant enrollment will be conducted by a well-trained team comprising two senior psychiatrists, one nurse, two full-time master’s students, and 20 psychiatrists who are motivated to refer eligible patients. The team has received targeted training to strengthen their understanding of the study rationale and its potential scientific and clinical significance. In addition, the project will be regularly promoted on the hospital’s official website, social media platforms, and in the outpatient lobby to support participant engagement. Finally, Tianjin Anding Hospital, the largest mental health institution in northern China, manages approximately 600,000 outpatient visits annually and has 1,400 inpatient beds; therefore, we anticipate that this optimized recruitment strategy will enable timely completion of the study.

### Study parameter/endpoint

#### Main study parameters/endpoints.

The primary outcome measure includes the change in the MADRS scores from baseline to the end of week 4.

#### Second study parameters/endpoints.

The secondary endpoints include the change of MADRS scores at the end of week 2, as well as the scores of the Self-Rating Depression Scale (SDS), Self-Rating Anxiety Scale (SAS), and Clinical Global Impression-Severity (CGI-S) at the end of weeks 2 and 4; the response rate and remission rate (defined as a 50% reduction in MADRS total scores from baseline and MADRS scores ≤ 10); and the presence of epileptiform activity in the EEG recordings.

#### Exploratory outcomes.

It will investigate alterations in EEG brain wave power and variations in the concentrations of peripheral biomarkers, including IL-6, TNF-α, IL-1β, glial fibrillary acidic protein (GFAP), S100 calcium-binding protein B (S100B), platelet-derived growth factor (PDGF), cluster of differentiation receptor-68 (CD68), CD74, CD14, and CD109. Additionally, it aims to detect the association between changes in MADRS scores and alterations in EEG parameters and/or concentrations of peripheral biomarkers. We will also conduct post-hoc exploratory analyses of peripheral biomarkers and EEG parameters to identify potential biological correlates of tACS response. These analyses are hypothesis-generating and were not powered for formal hypothesis testing.

#### Safety and tolerability.

Adverse events (AEs), vital signs, and electrocardiogram (ECG) parameters will be assessed. Serious adverse events (SAEs) are defined as any medical condition that results in death, is life-threatening at the time of occurrence, requires hospitalization, or leads to persistent or significant disability. Throughout the intervention, a trained researcher will be present at all times to monitor participants’ subjective sensations (including tingling sensations, phosphenes, dizziness, etc.) and to document any discomfort along with its intensity. An adverse event log will be established to record the time of occurrence, duration, severity, and the potential relationship to the intervention. Adverse events will be graded in a standardized manner using the Common Terminology Criteria for Adverse Events (CTCAE). Clear stopping rules will also be implemented: if an adverse event of Grade ≥ 2 occurs and is judged to be possibly related to the intervention, the stimulation for that participant will be suspended or terminated; in the event of serious adverse events such as skin burns, altered consciousness, or seizures, the intervention will be terminated immediately. If the same type of adverse event occurs in three or more participants, a safety review will be initiated.

#### Additional study parameters.

Information on name, sex, age, race and medications will be collected. The level of treatment resistance will be assessed via the DM-TRD, with a score greater than 12.5 serving as the threshold for defining TRD.

### Randomization, blinding and treatment allocation

A computer-generated random number table will be used to randomly allocate eligible patients to the active group or sham group at a 1:1 ratio. First, a statistician who is not involved in the trial will generate a randomization sequence via the PLAN procedure in SAS 9.4 software. Second, a nurse (also uninvolved in the trial) will place the group allocation results from the random number table into opaque, sealed envelopes. Each patient will receive a sealed envelope upon enrollment. To ensure rigorous allocation concealment, the group assignment information will be sealed in sequentially numbered, opaque envelopes, thereby precluding any arbitrary manipulation of the allocation sequence. Following sealing, the principal investigator will sign across the envelope closure, rendering any subsequent opening irreversibly detectable. Finally, on the first day of enrollment or the day the patient receives the first intervention, the researcher will open the envelope containing the group allocation information. Throughout the randomization process and the trial, both the active and sham groups, as well as the active and sham stimulation devices, will be labeled with letters A or B (only the device operator will know the letter assigned to the patient), ensuring that all trial personnel remain blinded to the type of stimulation (active or sham) administered or received. Additionally, the active and sham stimulation devices will be identical in appearance and sensory effects on the patient, so neither the patient nor the operator can distinguish between the two devices on the basis of appearance or the patient’s subjective feelings. Unblinding will occur after the statistical analysis of the study is completed. All procedures are strictly adhered to and are subject to periodic inspections by the regulatory authorities.

### Blinding assessment

All participants will be required to complete a blinding assessment using Bang’s Blinding Index (BBI) at the end of the trial to evaluate the success of blinding. The assessment consists of a brief questionnaire in which participants are asked to guess which treatment they believe they received (intervention or control), with an additional option of “do not know.” The BBI will be calculated separately for each treatment arm, with values close to zero indicating successful blinding.

### Study procedures

#### *Overview of the procedure*.

**Fig 1:**
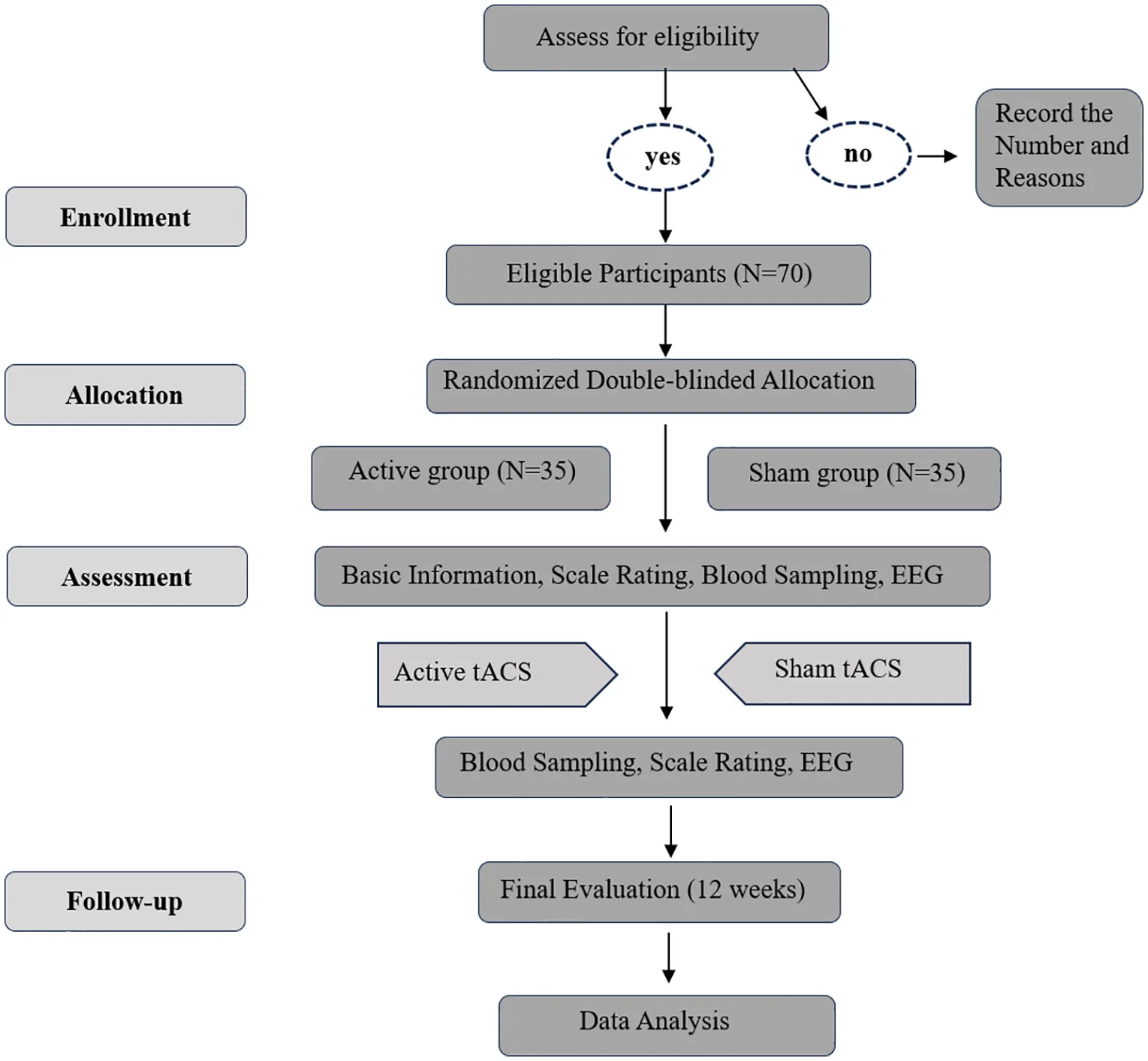
Trail flowchart.

Participants will receive a total of 20 sessions of tACS/sham intervention as add-on to antidepressant medication over a 4-week period, with interventions paused on Saturdays and Sundays, and will continue their original medication regimen during an 8-week follow-up period. Following completion of the screening procedures (DM-TRD, MADRS, and CRP), participants are enrolled and subsequently complete additional scale assessments and basic information collection on the same day (defined as the baseline). On the following morning, fasting blood samples (10 ml) are collected, and then the participants receive either tACS or a sham intervention, marking Day 1 of the 4-week intervention period. The day after completing all 20 sessions, participants will undergo a second round of blood sample collection (10 ml). At the same time points, information will be collected from the participants regarding EEG data and physical conditions such as heart rate and blood pressure to monitor their health status. The participants will complete four-round scale assessments, which will be conducted the day before the start of treatment, before the 11th treatment session, after completing all 20 treatment sessions, and after the 8-week follow-up. The specific time points and content of the scale assessments are detailed in Table 1. For an exploratory analysis, MADRS scores obtained on the day of the 11th intervention session, prior to the administration of active or sham tACS, will be utilized to investigate the potential early onset of treatment efficacy. Participants will receive standard care throughout the study, with the exception that no changes to their medication regimen are permitted during the study, except for benzodiazepines (BZDs). Antidepressants are limited to selective serotonin reuptake inhibitors (SSRIs) and serotonin-norepinephrine reuptake inhibitors (SNRIs), which must remain stable for at least two weeks prior to enrollment. BZDs may be prescribed during the first two weeks of antidepressant treatment to manage anxiety and for sleeping disturbances throughout the study, as deemed necessary by the treating physician. The dosage and duration of using BZDs and antidepressants will be recorded at each assessment during the trial and follow-up periods. The prescription of antipsychotic medications exceeding half of the recommended maximum dosage or for mood stabilization is prohibited. During the trial, the initiation of other neuromodulation interventions, including MECT, TMS, or tDCS, is prohibited. A total of 70 participants will be required to complete the study. See Fig 1. Trial flowchart for details.

The study will recruit 70 participants. Participants will undergo 20 sessions of tACS or sham intervention alongside their antidepressant medication over 4 weeks, with no sessions on weekends, followed by an 8-week follow-up period. Participants will complete basic information collection, blood sampling, and/or scale assessments at multiple time points. Note: tACS: transcranial alternating current stimulation; EEG: electroencephalography.

### Scale assessments

SDS: a self-reported inventory used to assess the severity of depressive symptoms.

SAS: a self-rating inventory for assessing the severity of anxiety symptoms.

MADRS: a clinician-administered scale designed to assess the severity of depression symptoms.

CGI-S: a clinician-rated scale used to assess the overall severity of a patient’s condition.

DM-TRD: a clinician-rated scale for measuring treatment resistance.

BBI: a questionnaire to evaluate the success of blinding.

### Active/sham tACS intervention

Participants sit comfortably in reclining chairs while receiving FDA/NMPA (National Medical Products Administration)-approved tACS (Nexalin Technology, Inc.), which is administered by trained physicians according to standardized instructions. A 4.45 × 9.53 cm electrode is placed on the forehead at Fpz, Fp1, and Fp2 following the 10/20 international placement system. Two 3.18 × 3.81 cm electrodes are placed on each mastoid. The tACS stimulation waveform includes a 180-second ramp-up period and a 12-second ramp-down period. The waveform is a square wave with an average amplitude of 15 mA, which is evenly distributed from the frontal region to the mastoid areas (the amplitude is reported as zero-to-peak). All participants receive 20 stimulation sessions at 77.5 Hz and 15 mA, whereas the sham condition delivers no active stimulation. One 40-minute session is administered each weekday (Monday to Friday) at a fixed time.

### Monitoring adherence

All active and sham tACS stimulations will be conducted in the hospital, strictly adhering to the study protocol and thoroughly documented. The concentrations of antidepressant and its metabolites will be measured exclusively in blood at various time points following the completion of the study. Adherence to antidepressants is further assessed through medical visit and medication records conducted by researchers. Additionally, we are confident in the patients’ adherence to treatment, supported by extensive collaboration with the patients’ guardians.

### Routine recordings

#### Basic information.

Name, age, gender, race

#### Physical examination.

The data, including height, weight, waist-hip ratio, and BMI, will be treated as confounding factors in the analysis; heart rate, blood pressure, and ECG will be used to monitor the participants’ health status.

#### Laboratory examination.

Blood (serum) will be collected at postbaseline and the day following the 4-week intervention (postintervention) and stored after centrifugation at −80°C. Samples will be used to assess the expression levels of biomarkers, which reflect glial cell functional status and the levels of inflammatory activity.

#### Blood.

Inflammation: IL-6, TNF-α, and IL-1β.

Glial cells: GFAP, S100B, PDGF, CD68, CD74, CD14, and CD109.

#### EEG.

Resting-state EEG recordings are obtained at post-baseline and again at post-intervention via a 64-channel EEG system (BrainProducts, Germany). The electrodes are placed in accordance with the international 10/20 system. Data are sampled at 5000 Hz, and electrode impedance is maintained below 10 kΩ. Each session consists of two conditions: participants first keep their eyes open for 5 min, followed by 5 min with their eyes closed. During the eyes-open period, participants are instructed to fix their gaze on a central crosshair.

### Data management

All data will be inputted into the EDC of “Six-dimensional Space” (https://h6world.cn/website/index.html).

### Auditing strategy

The sponsor will perform audits of the study in compliance with China’s Good Clinical Practice (GCP) guidelines. The frequency and procedures of these audits will align with relevant regulatory standards. All auditing activities will be carried out independently from the investigators to maintain objectivity and ensure regulatory compliance.

### Safety reporting

#### AEs.

Adverse events are defined as any undesirable experience that occurs to a participant during the study, regardless of whether it is deemed related to active/sham tACS. All adverse events, whether reported spontaneously by the participant or observed by the investigators or their staff, will be documented.

#### Serious adverse events (SAEs).

A serious adverse event is defined as any adverse medical occurrence or effect that results in death, is life-threatening at the time of the event, necessitates hospitalization or prolongation of an existing hospitalization, or leads to persistent or substantial disability or incapacity. Furthermore, any other significant medical event that does not directly cause death, poses an immediate life threat, or requires hospitalization may still be classified as an SAE if, in the judgment of a qualified medical professional, it could jeopardize the participant’s well-being or require intervention to prevent one of the aforementioned outcomes.

#### Reporting of SAEs.

The investigator is required to report any SAEs immediately to the sponsor and/or the designated Clinical Research Organization (CRO). In the event of an SAE, urgent unblinding of the patient must be conducted in the presence of at least two authorized personnel to ensure procedural integrity, followed by the administration of appropriate emergency medical treatment. Within a few hours of the event, the investigator must notify the State Food and Drug Administration (SFDA) of China by submitting the standardized SAE reporting form. Additionally, all relevant investigators involved in the study must be promptly informed of the occurrence.

#### Suspected Unexpected Serious Adverse Reactions (SUSARs).

Adverse reactions are defined as any untoward and unintended responses to an investigational product that are causally related to any administered dose. A Suspected Unexpected Serious Adverse Reaction (SUSAR) is identified when all of the following criteria are satisfied: (i) The event is serious, defined as an occurrence that results in death, is life-threatening, requires hospitalization or prolongation of existing hospitalization, or results in persistent or significant disability or incapacity. (ii) There is a reasonable possibility of a causal relationship between the event and the investigational product, indicating that the reaction is suspected to be harmful and unintended, and is attributable to the medicinal product under investigation, regardless of the administered dose. (iii) The adverse reaction is unexpected, meaning that its nature, severity, or outcome is not consistent with the applicable product information as described in the current Investigator’s Brochure (IB).

#### Reporting of SUSARs.

The procedure is same to the reporting of SAEs.

### Unblinding procedure

Routine unblinding will be conducted by the research assistant upon completion of the treatment period. Emergency unblinding is permissible only on a case-by-case basis for safety-related reasons, specifically when knowledge of the treatment assignment is essential for the clinical management of serious adverse events (SAEs). For such instances, sealed emergency unblinding envelopes are maintained in the medication storage room located in the inpatient ward, stored separately from the patches, and accessible 24 hours a day. In the event of a medical emergency, any qualified member of the medical team may open a sealed envelope to reveal the participant’s treatment allocation. All emergency unblinding events must be documented in the study records, including the justification and exact time of unblinding. The decision to initiate emergency unblinding rests with the local principal investigator.

### Withdrawal

#### Withdrawal of Individual subjects.

Participants may withdraw from the study at any time and for any reason without incurring any penalty or loss of benefits to which they are otherwise entitled. Additionally, the investigator retains the authority to discontinue a participant’s involvement in the study for urgent medical or safety-related reasons. All participant withdrawals will be classified as dropouts, and the reason for each withdrawal will be documented in the study records.

#### Defined criteria for withdrawal.

Participants will be withdrawn from the study under any of the following conditions:

1. Discontinuation of the investigational intervention (active or sham) for three consecutive days or a total of seven days, cessation of effective contraception, or occurrence of pregnancy.
2. Withdrawal of informed consent or the onset of SAEs related to active or sham tACS prompt study discontinuation, either at the participant’s request or at the discretion of the investigator.
3. Emergence of a manic episode during the study period.
4. Any modification to the participant’s antidepressant regimen during the trial, initiated either by the treating clinician or the participant.
5. The use of nonsteroidal anti-inflammatory drugs (NSAIDs) for a cumulative period exceeding seven days during the trial.

### Terminate participation

*Criteria for termination of subject participation:*

1. Withdrawal of her/his informed consent.
2. Intolerance to the study intervention: If the participant experiences adverse effects deemed intolerable or unacceptable, discontinuation of participation may occur, either at the participant’s request or following a joint decision with the treating physician.

### Statistical analysis

All statistical analyses will be performed using R software version 4.3.2. Descriptive statistics for continuous variables will be presented as means with SDs or medians with interquartile ranges (IQRs), as appropriate; categorical variables will be summarized as counts and percentages. All hypothesis tests are two‑sided, and a p‑value < 0.05 is considered statistically significant for the primary efficacy endpoint. The primary outcome is the change in MADRS total score from baseline to week 4, analyzed on the intention‑to‑treat (ITT) population (all randomized participants who have at least one post‑baseline MADRS assessment). Participants randomized but with no post-baseline MADRS assessments will be excluded from the primary analysis but included in the full analysis set (FAS) for descriptive purposes. A linear mixed‑effects model for repeated measures (MMRM) will be used, including fixed effects for treatment group (active tACS vs. sham), visit, and treatment‑by‑visit interaction; a random intercept for each participant; and pre‑specified covariates (baseline MADRS scores, baseline CRP level, and concomitant medications such as daily dose, duration of use, and drug class). An unstructured covariance matrix is assumed, and the Kenward‑Roger approximation will be used for denominator degrees of freedom. The primary contrast is the least‑squares mean difference in change from baseline to week 4. Missing data are handled under the missing at random (MAR) assumption, with the MMRM utilizing all available post‑baseline observed data via maximum likelihood estimation; sensitivity analyses (pattern‑mixture, tipping‑point) will test robustness to MAR violations. All outcomes other than the primary MADRS changes will be analyzed using analogous MMRM or appropriate generalized linear models, with results interpreted descriptively. The false discovery rate (FDR), a common issue in multiple comparison analyses, will be controlled using the Benjamini‑Hochberg procedure (threshold 0.05). For EEG power spectral density (PSD), a two‑step strategy will be applied: first, pre‑select frequency bands with >50% of measurements above the detection limit and a univariate association with MADRS change (p < 0.10); second, if needed, perform principal component analysis (PCA) to derive composite factors explaining ≥80% of the variance, with FDR correction across all EEG outcomes.

## Discussion

The relationship between heightened inflammatory activity and resistance to antidepressant treatments has been a central focus of recent research, emphasizing the complex interplay between the immune system and psychiatric disorders. One study on TRD revealed that peripheral IL-6 levels are associated with both the presence of TRD and the response to various antidepressant treatments [18]. This study specifically investigated the effects of bright light therapy (BLT) on serum IL-6 levels in TRD patients. While BLT reduced depression severity, it did not significantly alter IL-6 levels overall. However, responders to BLT exhibited increased IL-6 levels, suggesting that neuroinflammatory mechanisms may contribute to the etiopathogenesis of TRD and influence treatment response [18]. In another study involving unmedicated MDD patients, those with multiple failed treatment trials presented significantly higher plasma levels of TNF, soluble TNF receptor 2 (sTNF-R2), and IL-6 than those with fewer failed trials [19]. These findings support the hypothesis that elevated inflammatory activity might complicate antidepressant treatment outcomes and could serve as a biomarker for treatment responsiveness. Furthermore, our study revealed that an elevated baseline CRP level (0.85–10 mg/L) was a reliable indicator of a better response to antidepressant treatment combined with anti-inflammatory agents than to antidepressants plus placebo, although this trend disappeared when the baseline CRP level exceeded 1.76 mg/L (NCT02972398). The effect was most pronounced among participants with baseline DM-TRD scores greater than 12.5, indicating a higher level of treatment resistance (NCT02972398). It further highlights the potential of targeting inflammation or its downstream mediators as a strategy for improving treatment outcomes in TRD patients. tACS has emerged as a promising noninvasive neuromodulation technique for treating MDD. Several studies have supported its potential as an adjunctive therapy to conventional pharmacological approaches [20,21]. In particular, high-intensity tACS, specifically at 15 mA, has shown encouraging results in enhancing the antidepressant efficacy of standard treatments. One randomized controlled trial reported significant reductions in depression scores when 15 mA tACS was administered as an add-on treatment. This improvement was accompanied by a notable correlation between changes in alpha oscillatory power and reductions in depressive symptoms, suggesting that the antidepressant effects of tACS may be mediated by the modulation of cortical oscillations in the left frontal lobe [6]. The 15 mA tACS is considered to induce significant modulation of local field potentials in the hippocampus, amygdala, and insula under conscious conditions, with no seizure activity, cognitive decline, or serious adverse events observed across 11 SEEG-monitored patients. This finding establishes the first direct human evidence of safe and non-invasive deep brain stimulation at this intensity [22]. In the current study, we will further investigate the safety of 15 mA tACS in individuals with TRD and increased inflammatory activity.

To date, research on the antidepressant mechanisms of tACS has been limited, which has hindered the further development and widespread clinical application of tACS. Glial cells and neurons are known to engage in complex interactions that contribute not only to immune and inflammatory responses but also to neural functional states closely associated with depressive symptoms. These findings suggest a promising role for glial cells in the antidepressant mechanisms of tACS. To address this issue, the present study focuses on the efficacy and safety of high-intensity (15mA) tACS in treating patients with TRD and increased inflammatory activity. Specifically, the preference for high-intensity tACS arises from its potential to generate stronger electromagnetic effects, which may facilitate more extensive and profound neurophysiological changes to improve symptoms of TRD. Our previous research indicates that elevated baseline CRP levels can identify a high-inflammatory subgroup with a better response to anti-inflammatory treatment, enhancing sample homogeneity and increasing the reliability of the study findings. This approach aligns with the shift toward individualized treatment strategies. Additionally, using a dimensional approach to the TRD concept, rather than a definitional one, provides a more comprehensive and accurate assessment of treatment resistance [12]. For example, the DM-TRD model incorporates factors beyond antidepressant failure, including MECT, psychotherapy, and comorbid conditions. A score of >12.5 is chosen as the cutoff, based on our prior findings demonstrating favorable responses to NAC add-on therapy within this subgroup.

### Limitations

This design has several limitations. First, there remains a lack of consensus on the definition of TRD, particularly regarding the number of failed antidepressant trials required to establish treatment resistance, which may contribute to an imbalance between patient groups. To address this issue, we adopt the DM-TRD, a multidimensional inventory, rather than relying solely on the number of failed antidepressant treatments. However, the cutoff score of 12.5 is based on a single randomized controlled trial and requires further validation. Second, our protocol does not account for the duration of the current depressive episode, which has been identified as a potentially important factor in treatment resistance [23]. Third, the 20-session tACS intervention, although consistent with previous MDD studies [6], may be insufficient for addressing the symptoms of TRD associated with increased inflammatory activity. This limitation could increase the risk of false-negative outcomes. Additionally, although 15 mA tACS has been successfully applied in several clinical studies within the Chinese population, achieving significant results, the use of high-intensity electrical stimulation still carries a relatively high risk of adverse reactions, necessitating close monitoring during the intervention. Additionally, these adverse reactions may compromise the blinding of group assignments, leading to assessment bias. Therefore, we have arranged for two separate teams to evaluate safety (adverse reactions) and efficacy (symptom improvements).

## Conclusion

Despite these limitations, this study is expected to provide reliable evidence regarding the efficacy and potential mechanisms of 15 mA tACS in patients with TRD exhibiting elevated inflammatory activity. Understanding the role of inflammation in depression heterogeneity may inform mechanism-based treatments and explore personalized strategies tailored to individual biological profiles, particularly for difficult-to-treat patients.
